# Protein Chips for Detection of *Salmonella* spp. from Enrichment Culture

**DOI:** 10.3390/s16040574

**Published:** 2016-04-22

**Authors:** Palmiro Poltronieri, Fabio Cimaglia, Enrico De Lorenzis, Maurizio Chiesa, Valeria Mezzolla, Ida Barbara Reca

**Affiliations:** 1 CNR-ISPA, Institute of Sciences of Food Productions, via Monteroni km 7, 73100 Lecce, Italy; valeria.mezzolla@ispa.cnr.it (V.M.); Barbara.reca@ispa.cnr.it (I.B.R.); 2 Biotecgen, c/o Department of Biological and Environmental Sciences and Technologies, University of Salento, 73100 Lecce, Italy; f.cimaglia@biotecgen.it (F.C.); e.delorenzis@biotecgen.it (E.D.L.); m.chiesa@biotecgen.it (M.C.)

**Keywords:** antibody, biosensors, detection, food pathogens, identification, labelling, *Salmonella* spp.

## Abstract

Food pathogens are the cause of foodborne epidemics, therefore there is a need to detect the pathogens in food productions rapidly. A pre-enrichment culture followed by selective agar plating are standard detection methods. Molecular methods such as qPCR have provided a first rapid protocol for detection of pathogens within 24 h of enrichment culture. Biosensors also may provide a rapid tool to individuate a source of *Salmonella* contamination at early times of pre-enrichment culture. Forty mL of *Salmonella* spp. enrichment culture were processed by immunoseparation using the Pathatrix, as in AFNOR validated qPCR protocols. The *Salmonella* biosensor combined with immunoseparation showed a limit of detection of 100 bacteria/40 mL, with a 400 fold increase to previous results. qPCR analysis requires processing of bead-bound bacteria with lysis buffer and DNA clean up, with a limit of detection of 2 cfu/50 μL. Finally, a protein chip was developed and tested in screening and identification of 5 common pathogen species, *Salmonella* spp., *E. coli*, *S. aureus*, *Campylobacter* spp. and *Listeria* spp. The protein chip, with high specificity in species identification, is proposed to be integrated into a Lab-on-Chip system, for rapid and reproducible screening of *Salmonella* spp. and other pathogen species contaminating food productions.

## 1. Introduction

Most foodborne illnesses from foods consumed raw or cooked are caused by *Salmonella* species. *S. enterica* has evolved into a diverse range of pathogenic and non-pathogenic forms. Many serovars do not cause disease, some cause disease in both humans and farm animals, while others cause disease in humans but not in farm animals. *Salmonella* isolates have been sequenced at laboratories in Minnesota, Washington, and New York [1], providing a source for genomes comparison. *Salmonella* spp. was found in pasteurized milk and yogurt desserts in Greek child foods with a rate of 0.5% of analysed samples [2]. *Salmonella* spp. infiltrates plant leaves remaining persistent on salads, sprouts, seeds, peanuts and lettuce [3]. In recent *Salmonella* outbreaks in USA, the Centre of Disease Control recorded strains associated with spinach salads, ground meat, eggs, poultry products and peanut contamination. There is a need to rapidly identify and trace absence/presence of *Salmonella* spp., in processed or raw foods [4]. A pre-enrichment culture in liquid medium and cultures on selective agar plates are standard microbiological methods. These methods require 48 h to allow bacteria to grow in liquid culture and 48 h to grow on agar plate. Molecular methods detecting bacteria in culture at the end of exponential phase growth, can avoid the agar plate culture step. PCR has been extensively used in food safety tests for *Salmonella* [2,5,6,7]. The results of PCR reactions are detected at the end of the amplification process (end-point PCR, digital PCR) or during the amplification (Real-time or quantitative PCR) or by means of hybridisation onto oligonucleotide arrays [8]. 

Bacteria identification in food products is required at food production sites. *Salmonella* spp. have been cause of foodborne illnesses and require great attention. Very early detection and identification of bacteria, especially *Salmonella* spp., in food products will help the recalling of contaminated batches. Thus, improved methods allowing detection and identification in 24 h are required. 

With the aim to shorten the incubation time and identify contaminating bacteria in the same day, qPCR has been combined with immunomagnetic separation using AFNOR certified, proprietary lysis buffers and DNA purification kits. A new AOAC-RI validated 8-h protocol by Bio-Rad (Hercules, CA, USA), the iQ-Check Prep System, has brought the possibility to obtain *Salmonella* results within the same day of enrichment culture [9], allowing for fast results and rapid intervention in the food chain distribution. 

In the last years, biosensor based methods have been developed. There is a growing number of publications on rapid bacteria identification methods based on biosensors [10]. Label free and label-based biosensor methods have been reviewed recently, with variable results in sensitivity [11]. Electrochemical Impedance Spectroscopy (EIS) [12,13,14], has been applied in detection of *Salmonella* [15] and *E. coli* [16]. Surface Plasmon Resonance (SPR) [17,18,19,20] and quartz crystal microbalances (QCM) [21,22,23] in *Salmonella* detection produced results in the range of 10^4^–10^5^ colony forming units (cfu)/mL. Amperometric immunosensors have shown also the feasibility of detection of *Salmonella* and *E. coli* [24]. Chitosan-based multiwalls and carbon nanotubes [25], graphene-based electrical devices, carbon nanostructure-based field-effect transistors [25] and self assembled gold nanoparticles [26] have been developed in detection of *Salmonella* spp. However, the sensitivity reached by each method differs and it is difficult to compare the methods since some were applied to food matrix and some to cultured bacteria. Immunosensor methods have been combined with immunoseparation to concentrate the bacteria from enrichment cultures [27] prior to bacteria identification. Nano-sized immunomagnetic particles have been developed for specific separation and efficient concentration of *E. coli* O157:H7 [28] and in *Salmonella* detection combining magnetic nanoparticles (MNPs) and quantum dots (QDs) [29]. Sensors have been tested exploiting various labelling signals, fluorescent compounds, gold nanoparticles (AuNP), and CdSe/ZnS QDs labelling of antibodies to visualise *Salmonella* spp. and *E. coli* pathogens [30,31]. Devices using QDs were applied to detect *Salmonella* spp. on polycarbonate membrane [32], with enhanced sensitivity of the previously reported biosensor methods. 

Antibodies arrayed onto microarray glass slides as detection platforms [33] have been applied to identification of enterobacteria. In the antibody chip set up, a glass slide activated with epoxy-groups, for the binding of amino residues in a Schiff reaction, is spotted with capture antibodies [34] or using proteins with high affinity for a bacterial species [35]. Antibodies can be fluorescently labelled, or hybridised with labelled protein A or anti-IgG antibodies. Biosensors based on protein chips make use of laser scanners to read the fluorescence signal of excited molecules. Protein chips for detection of bacteria using fluorescent antibodies [36,37] showed variable detection limits, and there are no results obtaining a limit of detection lower than 10^3^ cfu/mL: in the case of *L. monocytogenes* the limit of detection was 10^4^ cfu/mL [19]. Advancements have been made in portable readers measuring 19 cm in length [38], reducing the complexity of the scanner. Advances in detection have been obtained through miniaturized non-cooled CCD cameras implementing lab-on-a-chip detection systems [39]. Fluorescence detecting webcams based on complementary metal oxide semiconductor (CMOS) can be integrated in these systems [40,41]. Smartphone based portable detectors are also a valuable method of image acquisition [42]. Immunosensors and protein chips are feasible to be implemented by automation, reduction of operation time, operator errors, equipment complexity and optimisation of cost-effectiveness. In the attempt to improve detection of bacteria and make the processing user friendly, protein chips may be integrated into a Lab-on-Chip (LOC). Presently the limiting bottlenecks for their integration are costs, portability, and automation [43,44,45].

In this study, with the aim to test the performance of antibody chips and the feasibility to miniaturize the technique using small volumes, protein chips for *Salmonella* were developed and detection methods and protocols were improved. We used immunoseparation to concentrate bacteria from enrichment culture, and tested either Pathatrix proprietary magnetic beads and in-house made antibody-bound magnetic beads. Immunoseparated bacteria were divided in two tubes. A bead-bound bacteria solution was used for DNA extraction and qPCR, to evaluate and count *Salmonella* spp. to confirm the results, while the second tube was used to perform the protein chip analysis. We tested different fluorescence labelling methods for bacteria, and selected a method for labelling directly the bacteria, that increased the sensitivity of the protein chip by ten folds. We developed protein chips containing 5 antibodies specifically binding to *Salmonella* spp., *E. coli, C. jejuni, S. aureus* and *L. monocytogenes*. We propose to apply the protein chip containing 5 different species-specific antibodies for bacteria analysis in multiplex. Finally, the protein chip method is ready to be integrated into a Lab-on-Chip device provided with miniaturised flux controls and detection devices.

## 2. Experimental Section

### 2.1. Bacterial Strains

*Salmonella* strains used in the study were isolated from meat products. A list of bacterial species and strains used in the experiments is provided in Table 1.

### 2.2. Immunoseparation of Salmonella spp. Through the Pathatrix Device Using Magnetic Bead-Ab Complexes

**Figure 1:**
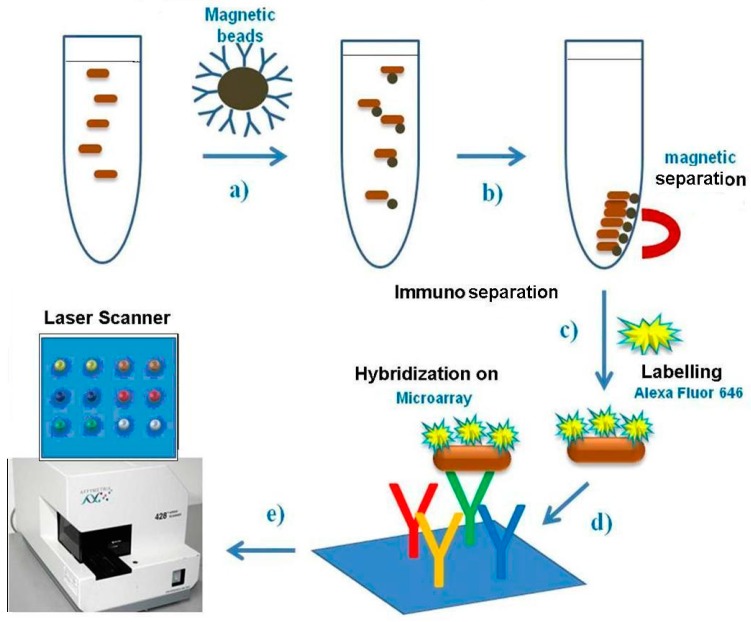
Scheme of protocol used, from Pathatrix immunoseparation to protein chip detection.

The Pathatrix Auto System (Life Technologies, Carlsbad, CA, USA) provided with anti-*Salmonella* antibody coated magnetic beads, AFNOR approved for immunomagnetic separation from a culture broth, was used for *Salmonella* spp. immunoseparation from 45 mL of enrichment culture. The medium with bacteria was loaded in a sterile 50 mL tube, to which 50 μL of magnetic beads were added, then a syringe was applied and the tube was adapted into the Pathatrix, a wash tube containing PBS was put in line with the device and the program was run until completion. The tube was detached from the magnet and transferred to the stand provided with a bottom magnet for bead collection. A scheme of the protocol describing the steps from immunoseparation to immunodetection is shown in Figure 1.

Magnetic beads activation. Antibodies were biotinylated using the biotin-XX-SSE method (Molecular Probes, ThermoFisher, USA) according to the procedure described by supplier. Two different antibodies were immobilised on magnetic beads indirectly through the biotin-streptavidin system: biotinylated anti-*Salmonella* antibody, and anti-Gram negative bacteria Lipopolysaccharide/Endotoxin Antibody (ab41201, Abcam, Cambridge, UK), were incubated with streptavidin magnetic beads (Pierce, ThermoFisher, Waltham, MA, USA), obtaining a working solution containing 1 mg/mL antibody bound to magnetic beads. Beads with three types of antibodies were tested into the bacteria immunoseparation tube, to which a magnet is applied on the tube bottleneck. After wash steps, bacteria were recovered in PBS. The beads were collected at the tube bottom, then volume was reduced down to 50 μL using a micropipette.

**Figure 2:**
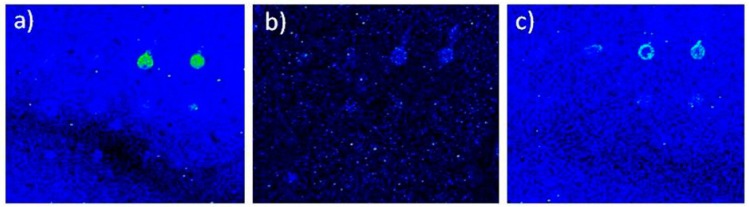
**Protein chip detection of bacteria immunoseparated using in-house made magnetic beads.** Fluorescence intensity showing the protein chip results of bacteria immunoseparated using three types of magnetic beads: (**a**) beads made using anti-*Salmonella* antibody indirectly bound by biotinylated group to streptavidin magnetic beads; (**b**) anti-Gram negative antibody; (**c**) Pathatrix beads. Scale: square size 4 mm.

Magnetic bead-bound *Salmonella* bacteria were counted by plating on Xylose Lysine Deoxycholate (XLD) agar for counting colony forming units. No differences in cell counts obtained using different immunoseparation beads were found at the bacteria level. Bacteria were denatured by thermal treatment before protein chip testing. However, when tested in protein chip analysis, the in house made streptavidin beads bound to biotinylated antibodies performed better than the Pathatrix beads, since protein chip result was less defined, due to a loss of bacteria during the transfer from the beads to the hybridisation solution (Figure 2).

### 2.3. Protein Chip for Salmonella Detection

Nexterion E glass slides, epoxy-activated for covalent binding of proteins were used (Schott AG, Mainz, Germany). Printing on the glass slides was made by SpotArray 24 (Perkin Elmer, Waltham, MA, USA) with deposition of 10 nL anti*-Salmonella* Abs (Abcam, Cambridge, UK), 0.5 mg/mL, in duplicate.

After deposition, the slides were left for 2 h in an incubation chamber at 60% humidity at room temperature. Then, the printed slides were kept dry in refrigerator until use. Before use, slides were saturated using TRIS 50 mM pH 7.4, 1 mM BSA and 100 mM cysteine for 10 min, washed with PBS + BSA (0.1%, *w*/*v*) and drained by centrifuging at 500× *g* for 2 min. Experiments were repeated three times for each experimental setting.

### 2.4. Immunoseparation Combined to Protein Chip Analysis

The protein chip was incubated with bacteria in a 60 μL incubation chamber (8 pad slide) (Whatman, Kerafast, 10486137) (Kerafast, Boston, MA, USA), using a chip clip slide holder (Whatman, Little Chalfont, UK), and kept 1 hr at room temperature. The slides were washed with TRIS buffer saline (TBS) with 0.1% Tween-20 (TBS-T), and 0.1% bovine serum albumin (BSA), under shaking, at 4 °C for 15 min.

In the detection of *Salmonella*, we used biotinylated antibodies and biotinylated anti-Salmonella aptamers (Bioapter, Madrid, Spain), in association to streptavidin bound Quantum Dots. The concentration of the biotinylated antibody was determined in a NanoDrop at 280 nm (Life Technologies, Carlsbad, CA, USA). Cadmium Selenide (CdSe) QDs (15–20 nm in size) with a maximum emission wavelength of 565 nm, shelled with ZnS and coupled to streptavidin, were purchased (Q10131MP) (Invitrogen, Life Technologies, Carlsbad, CA, USA). One μL of streptavidin-QDs was used to label 200 ng of biotinylated antibody in PBS.

The QD-antibody complex was added and left to hybridise on the protein chip in a 60 μL incubation chamber using a chip clip slide holder, and kept for 1 hr at room temperature, under dark. The slides were washed with TRIS buffer saline (TBS) with 0.1% Tween-20 (TBS-T), and 0.1% BSA, under shaking, at 4 °C for 15 min, then washed twice in PBS + BSA (0.05% *w*/*v*) for 5 min and dried in an Allegra centrifuge (Beckman, Brea, CA, USA) at 500 g for 2 min. The slides were analysed using a Laser Scanner 428 (Affymetrix, Santa Clara, CA, USA). Images were saved as TIFF or Bitmap files and analysed using ScanAnalyse.

### 2.5. Protein Chips for Detection of Bacteria

Protein chips were activated as previously described. *Salmonella* suspensions at various concentrations were incubated in a 60 μL incubation chamber using the chip clip slide holder for 1 h at room temperature. After incubation with the bacteria, a further incubation with fluorescent antibodies was performed as previously described. Then the slides were washed twice in phosphate buffer saline (PBS) + BSA (0.05% *w*/*v*), for 5 min and dried in an Allegra centrifuge (Beckman, Brea, CA, USA) at 500 g for 2 min. The slides were analysed using a Laser Scanner 428 (Santa Clara, CA, USA). Images were saved as TIFF or Bitmap files and analysed using ScanAnalyse. The resolution was not high enough, with sensitivity similar to previously reported values of 10^3^ cfu/mL. We tested various fluorescent dyes, with anti-*Salmonella* Alexa 555-conjugated antibody, biotinylated anti-*Salmonella* aptamers (Bioapter, Madrid, Spain) and BODIPY, a type of lipid binding dye. These protocols required an additional hybridisation step lasting 1.5–2 h until completion. 

The direct method (Cyanine 3-labelled antibody) did not provide enough sensitive detection, remaining in the range of 10^3^ cfu/mL as previosuly published [19]. Other fluorescent labelling methods were also tested, such as QDs.

Direct labelling of bacteria. In the experiments, bacteria were made fluorescent by direct labelling with Alexa 646. Bacteria, bound to the beads in 50 μL PBS, were thermally inactivated for 10 min, then were fluorescently labelled by addition of 1 μL Alexa Flour 646 (A32757, Invitrogen) (Life Technologies, Carlsbad, CA, USA) and incubated 2 hr at room temperature. Then the solution was centrifuged at 14,000 g × 5 min to eliminate the dye, bacterial pellet was washed, centrifuged and resuspended in 50 or 100 µL PBS and used for hybridisation on the protein chip. The scheme describing immunoseparation, direct labelling of bacteria and immunodetection is shown in Figure 1.

### 2.6. Protein Chip Sensitivity and Limit of Detection

Bacterial cells were hybridized on the protein chip, in a volume of 50 μL. After immunoseparation, bacteria were directly labelled, using the fluorescent Alexa-646 dye. 

**Figure 3:**
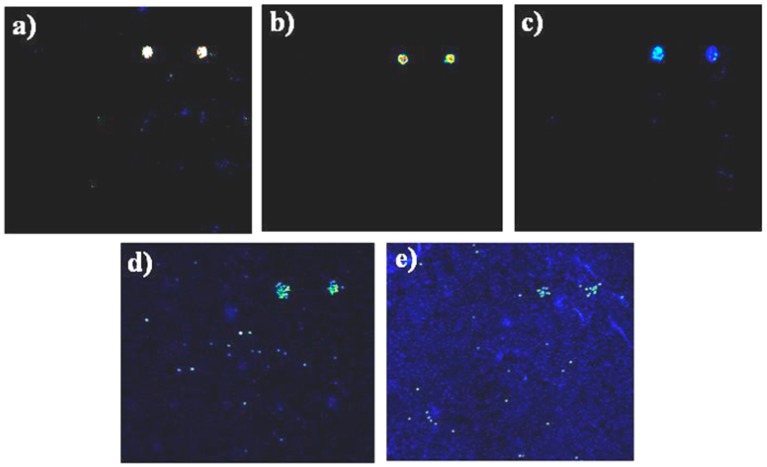
Assessing the limit of detection of protein chips using serial dilutions of *Salmonella* spp. Protein chips hybridised with serial dilutions of Salmonella cells after elution by thermal treatment of magnetic beads. Quantification of colony forming units performed in parallel by plating bacteria on XLD-Agar. Each sub-array was hybridised with a serial dilution of *Salmonella*, 10^6^ cfu/mL. (**a**) 10^5^ cfu/mL; (**b**), 10^4^cfu/mL; (**c**) 10^3^ cfu/mL; (**d**) and 100 cfu/mL; (**e**) Signals obtained by direct labelling of bacteria with Alexa 646. Experiments were repeated at least three times. Size of subarrays: 3 × 3 mm.

Bacteria in PBS buffer were deposited into the incubation chamber, and the chamber isolated using a clip slide holder, determining an internal volume of 60 μL, and incubated for 1 h in the dark at room temperature. A bacterial solution at known concentration was diluted in PBS. The concentration of bacterial dilutions was determined by plating on Xylose Lysine Deoxycholate (XLD) agar for cell counts and determination of colony forming units. In further experiments, serial dilutions of *Salmonella* spp. were released from magnetic beads after immunoseparation, and tested on the protein chip, to evaluate the limit of detection (Figure 3). Fluorescence intensity was high enough to allow the visualization and detection of 10^2^ cfu/mL of *Salmonella* bacteria as the lowest concentration detectable on the protein chip. Experiments were repeated at least three times.

### 2.7. Comparison of Sensitivity of Protein Chip Method with qPCR

*Salmonella* spp. cells were affinity-purified by Pathatrix immunoseparation using the Pathatrix® *Salmonella* spp. APS50 kit (Life Technologies, USA) and the lysis buffer (Cat. No. 4,480,724), included in the kit. To check the count of *Salmonella* in each experiment, duplicates of beads with bound *Salmonella* were tested, one for protein chip analysis and one by Real-time PCR.

Bacteria were lysed directly in the tube with magnetic beads, adding 100 μL lysis buffer and DNA purification was performed using a glass fibre column (GenElute, Sigma, St. Louis, MO, USA). Spectrophotometric determination of DNA was performed using a Nanodrop (Life Technologies, Carlsbad, CA, USA). PMB01A PATHfinder *Salmonella* spp. Assay (Generon, Modena, Italy) was used for the presence of *Salmonella* DNA positive controls. The species-specific primers were provided by Taqman *Salmonella enterica* kit (Applied Biosystems, Foster City, CA, USA).

A Premix was made by adding 10× buffer, primer/probe mix, H_2_O, then in each tube we added DNA, and Taq polymerase. The tubes were mixed, centrifuged and the content distributed in the microplate wells, covered by adhesive and read in the Applied Biosystems 7500 Real-time PCR instrument, using the following program for 35 amplification cycles: Step 1: 95 °C, hold 5 s; Step 2: 60 °C, annealing, hold 30 s, signal acquisition (FAM, JOE); Step 3: 72 °C extension for 45 s. 

By comparing the cycling ramp of control DNA and the corresponding cycling of DNA samples the concentration of the unknown DNA was calculated. The reference *Salmonella* DNA was used to produce a calibration curve at serial dilutions of starting concentration.

### 2.8. Manufacturing the Protein Chip for Detection of Bacteria in Multiplex

**Figure 4:**
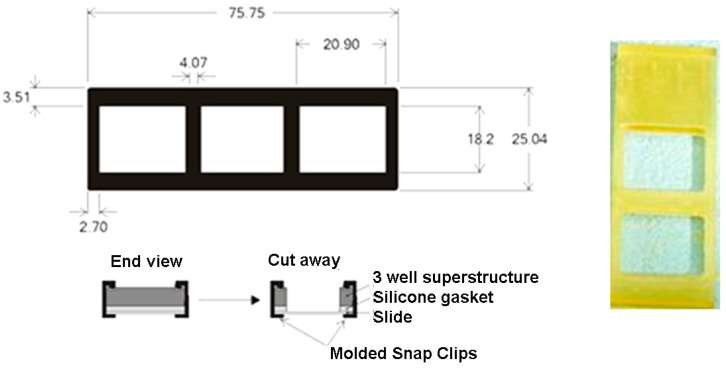
Scheme of printed slides in 2 subarrays, with slide holder closing gasket (digits in mm).

Nexterion E glass slides, epoxy-activated for covalent binding of proteins were from Schott AG, Germany. Printing on the glass slides was performed using a SpotArray 24 (Perkin Elmer, Waltham, MA, USA) as described in Section 2.3. A scheme of the printing instrument and the slide holder used in experiments is depicted in Figure 4. Antibodies used were purchased from Abcam, UK: anti*-Staphylococcus aureus* Ab (ab20920), anti-*Campylobacter jejuni* Ab (ab53909), monoclonal [LZH1] anti-*Listeria monocytogenes* antibody (ab11439), anti-*Salmonella* antibody (ab35156) polyvalent for “O” and “H” antigens, anti-*E. coli* antibody (ab137967). Negative control. Protein spots contained 6 different antibodies, printed in duplicate (12 spots), in volumes of 10 nL: anti-*L. monocytogenes* Abs, anti*-E. coli* Abs, anti*-Salmonella* Abs, anti*-S. aureus* Abs, anti-*C. jejuni* antibodies and a negative control (scheme in a grid of 12 spots, with two duplicates, distributed in two upper, two middle and two lower lanes). Antibodies were dissolved in PBS buffer (50 mM pH 7) at 0.5 μg/μL concentration. The Spot Array allowed deposition of 10 nL drops and miniaturisation into sub-arrays, with reduced spot size, arrayed in 8 sub-arrays. Each slide can hold up to 16 sub-arrays. After deposition, the slides were left to react for 2 h in an incubation chamber at 60% humidity at room temperature under dark. Then, the printed slides were kept dry in dark until use.

Before use, the slides were incubated for 1 h on a shaker at RT in a solution containing 0.1 M glycine and 1% *w*/*v* BSA in phosphate buffer saline (PBS) pH 7 and glycine 100 mM, for blocking the unreacted epoxy groups. Two wash steps with 1 × PBS, containing 0.1% Tween-20 were performed, and slides were dried by centrifugation at 500 g × 2 min.

Bacteria were cultured in liquid medium, up to exponential phase. Then, immunoseparation was carried out, in order to achieve similar concentrations. An aliquot of 10 μL magnetic beads-bacteria solution was used in each experiment, corresponding to about 10^4^ cfu/mL.

Slides were hybridised with five different bacteria samples, in the incubation chamber using 60 μL volumes, closed by the chip clip slide holder (Schleicher&Schuell, Whatman, Kerafast, USA) for 1 hr at room temperature. After the incubation, slides were washed with TRIS buffer saline (TBS) with 0.1% Tween-20 (TBS-T), then incubated with the detecting antibody in a similar volume. After removal of aspecifically bound antibodies by washing in TBS containing 0.1% Tween-20 and 0.1% BSA, under shaking, at 4 °C for 15 min, then washed twice in PBS + BSA (0.05% *w*/*v*) for 5 min and dried in an Allegra centrifuge (Beckman, USA) at 500 g for 2 min. The slide was scanned with a Laser Scanner 428 (Affymetrix, S. Clara, USA). Images were saved as TIFF or Bitmap files and analysed using ScanAnalyse. Experiments were repeated at least three times.

## 3. Results and Discussion

### 3.1. Immunoseparation of Bacteria in Enrichment Culture Using the Pathatrix Instrument

We tested various beads for bacteria immunoseparation from culture medium. 50 µL of magnetic beads activated with anti-*Salmonella* antibodies, were added to 40 mL of enrichment medium containing 10^9^ cfu/mL of *Salmonella* spp. culture. Kit Pathatrix containing anti-*Salmonella* antibodies, and biotinylated anti-*Salmonella* Abs conjugated to streptavidin magnetic beads made in house were used, as described in material and methods. Streptavidin beads linked to biotinylated anti-*Salmonella* antibodies, and Pathatrix proprietary beads were compared, especially in the efficient release of bacteria and subsequent visualization after hybridization on the protein chip.

Detection on the protein chip of bacteria captured using in-house made magnetic beads is shown in Figure 2 Streptavidin bead-bound biotinylated antibodies showed highest signal intensity in protein chip detection of bacteria. The experiments could have been affected by a series of cooperative factors (spots variability, local surface variability, different degree of blocking efficiency, backgroud noise and signal to noise ration variability). The experiment showed that Pathatrix beads performed well, but the protein chip images were of lower quality. On the contrary, the biotinylated antibodies and in-house made magnetic beads performed as well as the commercial beads, and showed to be better suited in inactivation of bacteria by thermal treatment, freeing easily streptavidin beads from the antibody-bacteria complexes. Therefore, all the experiments were conducted using anti-*Salmonella* antibodies conjugated to streptavidin beads. 

### 3.2. Protein Chip Detection of Serial Dilutions of Salmonella spp.

To test the protein chip and the labelling method most efficient in detection of *Salmonella* spp., various fluorescent labeling methods were compared. Direct labelling of antibodies or use of fluorescent secondary antibodies were not able to decrease the limit of detection below previous results, *i.e.*, 10^3^ cfu/mL [19]. The detection limit of the protein chip method using the direct labelling of bacteria with Alexa 646 was 100 bacteria/mL. This is an advancement on published results with a ten fold increase in sensitivity. Serial dilutions of *Salmonella*-bound beads were performed and tested on protein chips: the results of detection signals at various dilutions of bacteria are shown in Figure 3.

Figure 4 shows the scheme of the slide holder using a two subarray printed slide. However, up to 8 hybridizations were performed on a single slide. 

The limit of detection of protein chips combined with immunoseparation was 100 cfu/40 mL, with the possibility to process up to 250 mL of enrichment medium (1 cfu/2.5 mL). Immunoseparation from large volumes allows to bring the limit of detection independent from the working volume, that usually contains diluted numbers of bacteria, while the protein chip method requires to screen very low volumes on a small surface. Therefore, the protein chip combined with immunoseparation shows the potential of higher sensitivity and ability to detect the presence of *Salmonella* spp. in enrichment cultures at an early phase of growth, within the first 24 h.

In this study, we developed a protein chip for *Salmonella* spp. with a detection limit of 100 bacteria/mL. When combined with immunoseparation, the detection limit of the protein chip was independent from the volume of the antibody chip chamber. The lowest concentration of bacteria that can be detected on the antibody chip is 100 cfu. Since the volume of the enrichment culture used in this study was 40 mL, using the combination of two methods the limit of detection is 2.5 cfu/mL, with an increase in sensitivity of 400 fold. However, no limits are posed to the volumes that can be processed, even 250 mL of enrichment cutlure can be immunoseparated by Pathatrix using 5 Falcon tubes. 

The bead-bound bacteria solution was divided in two tubes, to process bacteria for detection using two different methods. 

### 3.3. Real-Time qPCR in Salmonella spp. Detection

A check on the number of *Salmonella* colony forming units in each experiment was performed using qPCR analysis. *Salmonella*-bound beads, collected after Pathatrix were divided in two tubes. One tube was used to perform protein chip detection, while the second tube was used to quantify bacterial DNA by RT-PCR.

After immunomagnetic separation, bacteria were lysed and DNA purified with a clean up column. The PCR analysis was used to confirm the protein chip positive and negative results.

PCR sensitivity in detection of *Salmonella* spp. DNA was determined using commercial Taqman microbiology kits (data not shown). PCR was able to detect between 1, 3 and 4 ng of genomic DNA (0.06 ng DNA/reaction (2 μL), using DNA quantified spectrophotometrically by lysis of *Salmonella* bacteria bound to beads. PCR analysis of *Salmonella* spp. bound to magnetic beads requires processing of bacteria with lysis buffer and DNA purification kits. Taking into account a volume of 50 μL for DNA purification during lysis step, the sensitivity of qPCR combined to immunoseparation is in the range of 2 genomic units/50 μL. While several methods have been applied for species identification after bacteria have reached the exponential phase in culture broth, there is a need to obtain the detection during the first 24 h of enrichment culture before bacteria reach the exponential phase of growth. Methods such as qPCR can provide results within the same day of enrichment culture [9]. It can be envisaged that also protein chip analysis combined to immunoseparation could be applied for rapid detection from enrichment cultures at early time points. 

### 3.4. Specific Detection of Five Food Pathogens using a Multiplex Device

Subsequently we performed hybridization experiments to test protein chips in multiplex analysis, and the efficiency in detecting each bacterial species without cross-reactivity.

**Figure 5:**
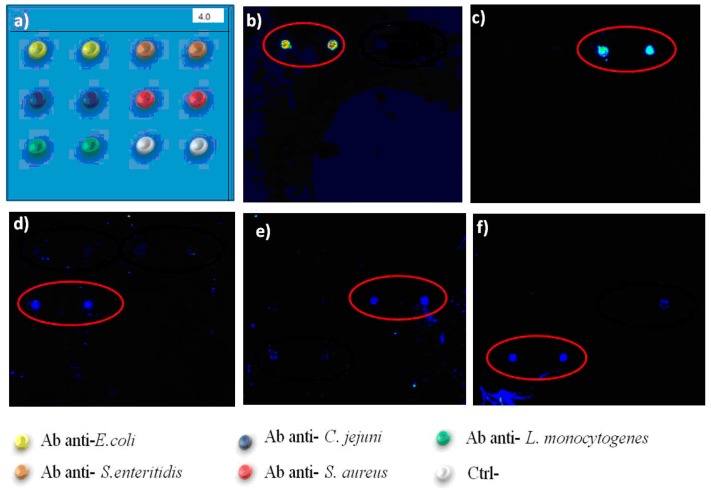
Assessment of performance of protein chips in detection of multiple bacterial species Left Upper line: Scheme of antibodies spotted in the *x*, *y* axis. Yellow: anti-*E. coli* Antibodies; orange: anti-*Salmonella* Abs; black: anti-*C. jejuni* Abs; green: anti-*Listeria monocytogenes* Abs; red: anti-*S. aureus* Abs. Size of the subarray area is 3 × 3 mm. *Salmonella* spp. were recognised by anti-*Salmonella* specific antibodies (**b**); *E. coli* (**a**) was detected in the anti-*E.coli* Ab spots. (**c**) *C. jejuni* was recognised by anti-*C. jejuni* Abs; (**d**) *Listeria monocytogenes* was recognised by anti-*Listeria* Abs; (**e**) *Staphylococcus aureus* was recognised by anti-*S. aureus* Abs. Experiments were repeated at least three times. Scale in mm. Each subarray area was 3 × 3 mm.

Figure 5 shows the screening of the protein chip in the identification of 5 bacterial species, for analysis in multiplex. In Figure 5, the scheme of the location on the protein chip of species-specific antibodies is shown for each of the bacterial species. High specificity of the protein chip device was observed in the case of two *Enterobacteriaceae*, *i.e.*, *Salmonella* spp. and *E. coli,* and in *Campylobacter* spp. In the case of *Campylobacter* spp., antibodies are not specific enough to discriminate at species or subspecies level, as also PCR methods have been questioned for the possibility to differentiate between *C. jejuni* and *C. coli* [46].

The immunoseparation allowed a first line of selection of each species with a first species-specific antibody, followed by the second round of specific binding, by capture of bacteria to the protein chip surface. Detecting antibodies may be used in the hybridisation step to increase the specificity, while this additional step requires longer times for completion. In this study, bacteria were directly labelled using Alexa 646, to avoid unnecessary slide processing steps, with a shortening of the time required for the protocol. The method used allows to avoid the use of fluorescent antibodies, with limited storage conditions and shelf-life and responsible for high background noise. The capture antibodies on the sensor surface assured the positioning of bacteria to the correct sub-array localization for all the five species tested. Each experiment showed the correct binding of bacteria to the corresponding antibody spot, without cross-reaction with different antibodies.

In conclusion, a protein chip was developed and tested in screening and identification of 5 common food pathogen species, *Salmonella* spp., *E. coli*, *S. aureus*, *Campylobacter* spp. and *Listeria* spp. The device for analyses in multiplex was efficient in differentiation of the 5 species, common in food contaminations. The multiplexing of the protein chip was limited to the identification of five bacterial species. A slide may contain up to 16 immunosensor subarrays and can be applied in screening of multiple assays in parallel. 

The protein chip showed an improvement over existing methods: it was more sensitive compared to previous results [19], is cost effective, more rapid since it avoids the last incubation with detecting antibodies, and accurate, since it has enhanced sensitivity and reproducibility. Therefore, it is envisaged that lab-on-chips based on bacteria detection could be developed in future at lower costs, at faster processing time and at convenient cost in respect to qPCR methods. Micro-reaction chambers, delivering small volumes through servo drives, high-precision mechanical components, and pumping systems with pulsation free fluid streams, syringe pumps, pump modules such as the Qmix modules (Cetoni GmbH, Germany) can be assembled to increase the automation of the performance of the device, enabling the setup of Lab-on-Chips [45] for *Salmonella* spp. and food pathogen detection, under automated control, reducing operation times and operator errors, providing rapid and easy-to-interpret results.

## 4. Conclusions

In conclusion, the biosensor method was applied to the detection of *Salmonella* spp. from enrichment culture, showing the feasibility of detection of bacteria at early phase of culture, similarly to the method validated on qPCR. A multiplex protein chip was developed and tested in screening and identification of 5 common food pathogen species, *Salmonella* spp., *E. coli*, *S. aureus*, *Campylobacter* spp. and *Listeria* spp. The protein chip for analyses in multiplex was efficient in differentiation of the 5 species, common in food contaminations. The multiplexing of the protein chip was limited to the identification of five bacterial species. The protein chip showed an improvement over existing methods. It is envisaged that lab-on-chips based on bacteria detection could be developed in future at lower costs, at faster processing time and at convenient costs, as an alternative to current qPCR methods, to detect bacteria at early phase of enrichment culture.

## Figures and Tables

**Table 1:** List of strains used in this work.

Species	Strain
*Salmonella enterica* subsp. *enterica*	ATCC13311
*S. enterica*	LBMM 10/CECT 952
*S. enterica*	LBMM 12/CECT 4141
*E. coli*	LBMM 288/CECT 4783
*E. coli*	LBMM 340/ CECT 4054
*Campylobacter coli*	LMG 6440T
*Campylobacter jejuni* subsp. *jejuni*	LMG 8841
*Staphylococcus aureus* subsp. *aureus*	LMG 22525
*Listeria monocytogenes*	LMG 23356
*Listeria monocytogenes*	LBMM 70/CECT 932
*L. innocua*	CECT 910, CECT 4030

Strains were kindly provided by David Rodriguez-Lazaro, Itacyl, Valladolid, Spain (CECT collection) and by Federico Baruzzi, CNR-ISPA, ITEM strain collection, Bari, Italy.
